# The Relationship Between Organization in Time, Executive Functions, and Quality of Life in Adult ADHD

**DOI:** 10.3390/brainsci15121262

**Published:** 2025-11-25

**Authors:** Nufar Grinblat, Sara Rosenblum

**Affiliations:** Laboratory for Complex Human Activity and Participation (CHAP), Department of Occupational Therapy, Faculty of Social Welfare & Health Sciences, University of Haifa, Haifa 3103301, Israel; nufarlev@gmail.com

**Keywords:** adult ADHD, organization in time, executive function, quality of life

## Abstract

**Background/Objectives.** Despite the importance of organization-in-time ability for adults’ daily performance, knowledge about this ability in adults with attention-deficit/hyperactivity disorder (ADHD) remains limited. This study aims to compare organization-in-time abilities and quality of life (QoL) in adults with ADHD versus controls and examine the association and predictive relationship between this population’s organization-in-time and executive function (EF) abilities and their QoL. **Methods.** Participants were 69 adults with ADHD and at least one EF deficit according to the Behavior Rating Inventory of Executive Function-Adult version (BRIEF-A) and 52 matched neurotypical controls. Besides the BRIEF-A and demographic questionnaire, all participants completed the Time Organization and Participation Scale and Adult ADHD QoL questionnaire. The study was approved by the institutional ethics committee, with written informed consent obtained from all participants. **Results**. Compared with controls, adults with ADHD demonstrated significantly poorer organization-in-time ability and QoL. Within the ADHD group, significant correlations were found between organization-in-time and EF abilities (*r* = −0.39 to −0.50, *p* < 0.01). The group (ADHD vs. control) explained 51.7% of the variance in total QoL. Beyond this, metacognitive abilities (BRIEF-A Metacognitive Index) accounted for an additional 15.1%, and organization-in-time domains contributed 10.8% of the variance in predicting total QoL. **Conclusions.** Identifying deficits in time-organization abilities and EFs and their association with lower QoL among adults with ADHD has empirical and clinical implications. Such identification and the development of targeted intervention programs are essential for improving QoL in this population.

## 1. Introduction

Attention-deficit/hyperactivity disorder (ADHD) is a neurodevelopmental condition defined by patterns of persistent age-inappropriate inattention, hyperactivity, and impulsivity. The Diagnostic and Statistical Manual of Mental Disorders (5th ed., text rev.; *DSM-5-TR*) [1] states that childhood ADHD symptoms may continue into adulthood, with a prevalence rate of 2.5%. However, ADHD is currently underdiagnosed and undertreated in adults, leading to a chronicity of symptoms and impairments [2,3]. Further, adults with ADHD are at increased risk for other neurodevelopmental conditions, such as specific learning disabilities (SLD) [4,5]. Building on methodological frameworks previously applied in a related study [6], the present work focuses on adults diagnosed with ADHD.

The *DSM-5-TR* definition [1] and other evidence, for example, Ref. [7], emphasize that ADHD is characterized by behavior patterns leading to difficulties in everyday performance. Understanding the health, cognitive, and activity characteristics of adults with neurodevelopmental disorders is crucial for clarifying their daily needs and overall quality of life (QoL) [8]. Examining these characteristics from the individual’s subjective perspective is also important because self-perceived experiences may reveal functional and emotional aspects of daily life that are not fully captured by objective assessments [9,10].

Given that an individual’s organization-in-time ability is closely linked to their daily function and participation abilities [11], it may represent an important factor underlying the daily challenges experienced by adults with ADHD.

Organization in time reflects individuals’ capacity to manage multiple goals within time constraints through planning, executive control, and time perception [12,13], representing the ability to organize daily activities in a timely and effective manner [11]. Although clearly related to everyday functioning, this ability has received little research attention among adults with ADHD. Prior studies focused mainly on individuals with SLD or the general population [6,11,14]. Therefore, the present study aims to clarify the time-organization challenges characterizing adults with ADHD. These time-related abilities rely on cognitive control processes, linking organization-in-time to the broader framework of executive functions (EFs).

Organization and time-management skills and time-perception ability directly involve executive functioning abilities [15,16]. According to the hybrid neuropsychological model [17,18,19] and recent literature [20,21], EFs are core defining factors in ADHD. The term *EFs* refers to a set of higher-level cognitive functions that is the underlying mechanism required to perform and participate in self-directed, complex, and nonroutine activities in varied situations and environments effectively, for example, Ref. [22]. They are composed of two close but separate executive abilities: metacognitive and motivational–emotional [23,24].

Given the extant literature, a relationship between EFs and organization-in-time of daily activities appears to be a reasonable hypothesis. Supporting this assumption, evidence has shown that adults with ADHD tend to prefer small, immediate rewards over larger, delayed rewards—a tendency associated with daily EF impairments [25]. These impairments may, in turn, influence organization-in-time of daily activities. Nevertheless, this relationship has not yet been comprehensively examined in adults with ADHD, only in those with SLD [6,14]. Further, the few researchers who investigated this association within the ADHD population examined the time dimension in a laboratory setting, without considering its real-life daily performance context, for example, Ref. [26].

Evidence from ADHD populations underscores the importance of integrating individuals’ self-perceived cognitive functioning through ecologically valid self-report assessments that capture ADHD-related functioning in real-life contexts [9,10,27,28]. Accordingly, the present study used self-report instruments to assess EFs and organization-in-time abilities, providing insight into how adults with ADHD experience and manage their everyday performance. Understanding this interplay in real-life contexts is crucial for clarifying how cognitive and temporal organization abilities contribute to broader outcomes such as QoL among adults with ADHD [29,30,31,32].

Quality of life, a multidimensional construct, primarily concerns patients’ personal evaluations of their lives regarding global health, handicaps or impairments, and daily living activities. Additionally, it encompasses nonmedical aspects of a person’s life, such as satisfaction with social, educational, and occupational functioning [32]. Because QoL reflects individuals’ subjective perceptions of their well-being and daily functioning, its valid assessment typically relies on self-report measures, which capture personal experiences that objective indicators alone cannot reflect [33,34].

There appears to be a consensus that adults with ADHD experience lower QoL than those without, and that this disadvantage often persists into adulthood, affecting education, employment, and health outcomes [35,36,37,38]. Difficulties in executive functioning and organization-in-time may contribute to this reduced QoL by limiting daily and occupational performance. For instance, organization-in-time abilities are necessary to work independently, complete long-term projects, and maintain consistent daily routines [11,39]. Moreover, EF deficits are strong predictors of functional impairments in adults with ADHD, for example, Refs. [40,41]. However, previous research has primarily focused on the association between EF and QoL [29,30], exploring the relationship between organization-in-time abilities and QoL only among the general population and adults with SLD [6].

Therefore, despite the importance of organization-in-time ability to adults’ daily performance, for example, Ref. [11], knowledge about this ability among adults with ADHD—its interaction with EFs and its potential contribution to QoL—remains limited. Previous findings among adults with SLD demonstrated similar associations between these variables [6], yet ADHD presents unique characteristics [1], warranting focused investigation within the ADHD population.

In addition, understanding how individuals interpret and respond to their cognitive and daily experiences is essential for capturing ADHD-related functioning [8,9]. This understanding is particularly relevant for QoL, a construct inherently defined by individuals’ subjective perceptions of well-being and daily functioning [33,34]. Therefore, self-report measures are crucial: They not only capture these personal perceptions but also provide ecologically valid information that complements objective assessments and illuminates how adults with ADHD experience and manage their daily functioning in real-life contexts [27,28].

Accordingly, the present study aimed to (a) compare the organization-in-time of daily activities and QoL between adults with ADHD and matched controls, and (b) examine the associations and predictive relationships between organization-in-time of daily activities, EFs, and QoL in adults with ADHD. Thus, this study addresses a gap in the literature by offering a novel conceptual integration of EFs and organization-in-time abilities to better explain QoL. By utilizing ecologically valid self-report measures, the study captures the subjective, real-life challenges of adults with ADHD, providing a unique person-centered perspective on their daily functioning.

## 2. Materials and Methods

### 2.1. Procedure

This study is part of a larger, previously reported study [42]. The data were collected between March 2017 and April 2018 following ethical approval. People from the community, with and without ADHD, were invited via email or social networks to participate in the study. The recruitment of a community-based convenience sample reflects real-world variability and enhances ecological validity. Those who contacted the researcher for inclusion in the research group were asked to present proof of a formal medical ADHD diagnosis by an expert neurologist, psychiatrist, or family doctor based on the *DSM-5* criteria.

Data were collected through a secure online platform that ensured anonymity and standardized administration, enhancing ecological validity by allowing participants to respond in their natural environments. After signing informed consent, all participants completed self-reported questionnaires (details follow) to confirm inclusion criteria and symptoms consistent with ADHD [43]. After confirming their eligibility, participants were sent a link to complete the study questionnaires (dependent variable evaluations; description follows). The lead researcher was available by telephone for assistance if needed, and all participants received a voucher as compensation for their time and effort.

### 2.2. Participants

A convenience sample of 121 adults from Israel participated in the study. The inclusion criteria for the entire sample were (a) aged 20 to 46 years, (b) working at least 3 months at the same place, and (c) read and write Hebrew fluently. People with motor or neurological disabilities, chronic diseases, or significant injuries that could affect participation and performance were excluded. A research-group-specific inclusion criterion was a formal medical diagnosis of ADHD by an expert neurologist, psychiatrist, or family doctor based on the *DSM-5* criteria. Symptoms consistent with ADHD were also confirmed by either the Adult ADHD Self-Report Scale (ASRS) version 1.1 [43] final score or medium-to-high probability of ADHD according to the Brown Attention-Deficit Disorder Scales (BADDS) [44]. Further, the research-group participants had at least one EF deficit according to the Behavior Rating Inventory of Executive Function-Adult version (BRIEF-A) [45]. Control-group members were without both ADHD (per either the ASRS final score or low probability of ADHD per the BADDS) and a learning disability diagnosis (based on their self-report). Further, they had no EF deficit (per the BRIEF-A). The inclusion and exclusion criteria were designed to minimize confounding factors while maintaining ecological validity by preserving the natural variability typical of community-based adult ADHD samples. The final sample included 121 adults—69 with ADHD and 52 without ADHD. The two groups were matched for gender, age, education level, and socioeconomic status (α > 0.05).

### 2.3. Instruments

The study used well-validated, standardized self-report instruments selected for their psychometric strength and ecological validity. Self-reports were used to capture participants’ subjective perceptions regarding study variables, providing insights that complement performance-based neuropsychological measures. This approach offers high ecological validity by reflecting real-world functioning [27,28]. It is particularly relevant for assessing QoL, a construct inherently defined by personal evaluation of daily well-being and functioning, for example, Ref. [33].

#### 2.3.1. Participants’ Background Characteristics

We used a self-report sociodemographic questionnaire to gather data related to the participants’ sociodemographic status (13 questions), diagnosis (five questions), and current employment status (nine questions).

#### 2.3.2. Organization-in-Time

The Time Organisation and Participation Scale (TOPS) [11] is a self-report scale that assesses the individual’s perceived ability to organize daily life tasks on time. The questionnaire comprises 34 items in four parts (domains): A, daily task performance at an appropriate pace (20 items); B, the individual’s success in organizing a whole day or a specific period satisfactorily (five items); C, the frequency of emotional responses following disorganization in time (seven items); and D, the influence of change in routines and various stimuli on the individual’s organization of time abilities (two items) [11]. In all domains, lower scores indicate a higher risk of difficulties in organization-in-time and participation-in-time in daily tasks. Because Domain D items are used for clinical purposes, we did not statistically analyze them in this study. Participants with a mean final score of less than 3.16 were considered at risk for difficulties in on-time organization of daily life tasks [11]. The TOPS was found to have high internal consistency, good content, face and concurrent validity, and discriminant validity among adults with and without SLD [6,11,14]. The TOPS showed high internal consistency and good content, face, concurrent, and discriminant validity among adults with and without SLD [6,14], and further evidence supports its validity in nonclinical populations [11].

#### 2.3.3. Executive Functions

The BRIEF-A (Psychological Assessment Resources, Lutz, FL, USA), a standardized, well-validated, reliable, and ecological self-report, captures adults’ views of their executive functioning in everyday environments, for example, Ref. [45]. The score includes a global executive composite (GEC) index that represents the overall EF score, composed of a behavioral-regulation index (BRI) score and a metacognition index (MI) score with nine nonoverlapping score scales. Raw scores are transformed into *t* scores according to age and gender norms: scores of 65 or more (*SD* > 1.5) for each index or scale indicate a clinical deficit. Results of the BRIEF-A Hebrew version supported its internal consistency, structure, and discriminant validity among adults with ADHD and SLD [6,14].

#### 2.3.4. Quality of Life

The Adult ADHD Quality of Life Questionnaire (AAQoL) [46] is a self-report that measures the QoL of adults with ADHD by focusing on four domains: life productivity, psychological health, relationships, and life outlook. The AAQoL consists of 29 items rated on a 5-point scale for frequency of occurrence, which yields a total score and four subscale scores (reflecting the four domains). Raw scores are transformed to a scale ranging from 0 to 100, with higher scores indicating better QoL [47]. The AAQoL has good internal consistency, construct validity, and test–retest reliability and discriminates between groups with and without ADHD, for example, Refs. [48,49].

### 2.4. Statistical Analysis

We performed all statistical analyses using IBM SPSS (version 25) with a significance level of *p* < 0.05 and calculated descriptive statistics to describe the sample. Statistical procedures were selected to test theoretically driven hypotheses and examine group-level differences and predictive associations, rather than exploratory or data-driven patterns. Power analyses (G*Power 3.1; Multiple analyses of variance [MANOVA]-Pillai’s trace) showed that detecting small-to-medium effects, f^2^(V) = 0.15, α = 0.05, 1 − β = 0.90, requires *N* ≈ 108. Therefore, the achieved sample (*N* = 121) provided sufficient power, retaining 1 − β ≈ 0.80 for smaller effects, f^2^(V) = 0.10, Pillai’s V ≈ 0.09. The demographic variables of the two groups were compared using chi-square tests. A MANOVA was applied to analyze differences in dependent variables between the groups, and subsequent univariate analyses of variance (ANOVA) were applied to find the source of significance across the scale components. Pearson correlations were used to examine the relationships between dependent variables among the research group, and predictive regression models were applied to examine the predictive relationships among the variables.

## 3. Results

### 3.1. Sociodemographic Characteristics

A detailed description of the participants’ sociodemographic characteristics has been previously reported [42]. Within the ADHD group, 17 participants reported an additional SLD (*n* = 17), and eight reported psychiatric comorbidities (depression, *n* = 3; posttraumatic stress disorder, *n* = 2; anxiety, *n* = 3). The gender majority in both the ADHD group (*n* = 69) and the control group (*n* = 52) was women (65.2% and 69.2%, respectively). Ages ranged from 21 to 46 years. No significant group differences were found in age, gender, socioeconomic status, familial status, or education level. Furthermore, participants in both groups had similar occupational characteristics [42].

### 3.2. ADHD-Group EF Background Characteristics

As mentioned, the inclusion criteria for the research-group participants were at least one EF deficit according to the BRIEF-A [45]. Analyzing the EF profile of research-group participants revealed general executive difficulties (80.9%) and metacognitive (80.8%) and behavioral regulation (63.2%) deficits among most participants, with higher mean scores (more deficient EF) in the MI scales (*M* = 75.15, *SD* = 10.36) than in the BRI scales (*M* = 67.94, *SD* = 10.06).

### 3.3. Between-Group Differences in Organization-in-Time Abilities (TOPS) and QoL (AAQoL)

#### 3.3.1. Organization-in-Time

The MANOVA indicated significant group differences for the TOPS Domains A, B, and C, *F*(3,116) = 58.54, *p* < 0.001, η^2^ = 0.60. As presented in Table 1, the subsequent univariate ANOVA revealed that the ADHD group participants had significantly lower mean scores in those three TOPS domains (*p* < 0.001). Thus, compared to adults without ADHD, participants with ADHD rated themselves with a slower pace of activity performance (Domain A), more performance difficulties (Domain B), and more emotional responses following their inappropriate performance in time (Domain C).

#### 3.3.2. Quality of Life

Significant group differences were found for the AAQoL total score, *t*(117) = −11.14, *p* < 0.001. A MANOVA indicated significant group differences for the AAQoL subscales, *F*(4,114) = 53.41, *p* < 0.001, η^2^ = 0.65. As presented in Table 2, the subsequent univariate ANOVA revealed that ADHD group participants had significantly lower mean scores in all AAQoL subscales (*p* < 0.001). That is, compared to the adults without ADHD, the adults with ADHD rated their QoL lower. Observed group differences were large, with univariate η^2^ values ranging from 0.39 to 0.65 across the TOPS and AAQoL domains and Cohen’s *d* ≈ 2.17 for the total AAQoL score (Table 1 and Table 2), well above the small-to-medium effects assumed in the power analysis.

### 3.4. Correlations Between EF Background Characteristics (BRIEF-A) and Organization-in-Time Abilities (TOPS) Among Adults with ADHD

Pearson correlations revealed significant medium-to-high correlations between GEC scores (BRIEF-A) and all TOPS domains (A: *r* = −0.46, *p* < 0.01; B: *r* = −0.43, *p* < 0.01; C: *r* = −0.39, *p* < 0.01); MI scores (BRIEF-A) and TOPS Domains A and B (A: *r* = −0.55, *p* < 0.01; B: *r* = −0.58, *p* < 0.01); and BRI scores (BRIEF-A) and TOPS Domain C (*r* = −0.50, *p* < 0.01). These results associate EF difficulties with time-organization deficits among adults with ADHD.

### 3.5. EF (BRIEF-A) Background Characteristics and Organization-in-Time Abilities (TOPS) as Predictors of QoL (AAQoL)

Regression analysis, as presented in Table 3, revealed that the group accounted for 51.7% of the total QoL (AAQoL), *F*(1,119) = 127.19, *p* < 0.001. Metacognitive abilities (BRIEF-A, MI) accounted for more than 15.1%, *F*(1,118) = 53.52, *p* < 0.001; emotional responses following unsuccessful organization of time (TOPS Domain C) accounted for 9%, *F*(1,117) = 43.49, *p* < 0.001; and satisfactory daily time-organization performance (TOPS Domain B) added another 1.8% of the variance to the prediction of total QoL (AAQoL), *F*(1,116) = 9.04, *p* < 0.01. Appropriate organization pace (TOPS Domain A) was not found to be a predictor of QoL. Together, these three variables predicted 25.9% of the variance of participants’ perceived QoL (AAQoL) beyond the contribution of group membership. It should be noted that the regression models presented here are associative and explanatory. Consistent with the cross-sectional design, they explain statistical variance rather than causal or temporal prediction.

## 4. Discussion

This study examines the organization-in-time of daily activities, EFs, and their associations with QoL in adults with ADHD compared to controls. Beyond these associations, it provides an ecologically valid perspective on how adults with ADHD perceive their daily cognitive functioning, deepening understanding of their real-life challenges. Consistent with this ecologically oriented approach, including ADHD participants with common neurodevelopmental and psychiatric comorbidities, for example, Refs. [4,5], reflects the real-world heterogeneity of this population and thus supports the generalizability of the findings.

### 4.1. Executive Function Characteristics in Adults with ADHD

Although the inclusion criteria for participation in the research group required at least one EF deficit (per BRIEF-A) [45], the results of the background characteristics analysis indicated a high percentage of participants with global executive deficits (per BRIEF-A GEC). This finding supports Barkley’s [17,18,19] findings regarding the central role of EF deficits in ADHD and further evidence indicating a very high prevalence of clinically significant EF deficits among adults with ADHD [20,21,29,50,51]. Specifically, the present findings reveal a pronounced imbalance between metacognitive and behavioral-regulation executive components (BRIEF-A MI and BRI, respectively) among the adults with ADHD, with greater metacognitive deficits in this population. This pattern aligns with previous research [14] and reinforces the evidence for the centrality of metacognitive impairments in adult ADHD. As Stern and Maeir [48] noted, this profile highlights the specific executive challenges that adults with ADHD face, which may differ from the greater behavioral-regulation difficulties observed in children with ADHD. As these metacognitive deficits have a major impact on this population’s daily function [52], accurate identification of EF characteristics in adults with ADHD will enable a more comprehensive understanding of their challenges in daily activities and allow for deeper analyses of how these difficulties interact with other ADHD features.

### 4.2. Between-Group Differences in Organization-in-Time and Quality of Life

The current findings also extend previous evidence from adults with SLD [6,14], showing that similar organization-in-time difficulties also characterize adults with ADHD. These individuals reported performing daily tasks at a slower pace, organizing their days less satisfactorily, and experiencing more frequent emotional responses following unsuccessful time management. These findings provide a basis for examining how difficulties in organizing daily activities in time may relate to broader aspects of QoL in adults with ADHD. In line with these findings, and consistent with previous evidence indicating reduced QoL among adults with ADHD [29,30,31,35,36,37,38], the present study also found lower QoL across all domains compared to controls. Notably, the total mean AAQoL score (*M* = 58.0, *SD* = 12.37) closely matched that reported by Brod et al. [47] among adults with ADHD (*M* = 60.3, *SD* = 19.00), reinforcing the robustness of this pattern.

Whereas prior research among children with ADHD and EF deficits identified impairments mainly in social QoL domains (e.g., family and peer relations) [53], our results indicate broader reductions across all QoL domains in adults with ADHD. Lee et al. [54] suggested that age may moderate QoL, with older participants reporting lower satisfaction with life—a finding that could help explain this more generalized impact in adults. Together, these results underscore the need to understand better how organization-in-time abilities and QoL interact in adults with ADHD.

### 4.3. Associations Between Executive Functions and Organization-in-Time

The significant correlations between EFs and organization-in-time abilities found in this study are consistent with previous research linking EF to time perception and time-management skills in adults with ADHD [15] and studies showing similar patterns among adults with and without SLD [4,14]. Beyond replicating these associations, the present findings emphasize the importance of assessing EF within real-life contexts. Goldin et al. [55] noted that understanding how executive processes operate in everyday functioning and real-world contexts is crucial. By applying this perspective, the present study provides insight into how executive difficulties are expressed in daily performance, extending previous laboratory-based findings. Among these executive components, metacognitive abilities appear particularly relevant because they are central in organizing and monitoring daily activities.

Metacognitive abilities enable individuals to regulate, monitor, and control their cognitive processes before responding [56] and to organize their daily activities in time. Accordingly, the significant correlations observed in this study between metacognitive abilities and timely organization of daily activities (TOPS Domains A and B) are consistent with this theoretical framework. Barkley’s [17,18,19] model further explains the observed association between executive emotional control and the emotional responses that follow disorganization in time (TOPS Domain C). According to Barkley’s model, deficits in emotional control may reduce one’s ability to generate and maintain the emotional state required for goal-directed behavior, which may lead to frequent emotional reactions following disorganization in time [14]. Beyond its theoretical contribution, these findings offer practical insights into how distinct executive components relate to specific daily time organization domains, informing a more precise understanding of the daily performance challenges experienced by adults with ADHD. Given the central role of these components in daily functioning, their combined influence is likely to extend to broader outcomes such as perceived QoL.

### 4.4. Predictors of Quality of Life: An Integrated Perspective

Building on this framework, the study examined how these mechanisms relate to the reduced QoL commonly observed in adults with ADHD. The findings underscore that group membership (ADHD vs. control) had a substantial impact on QoL, reinforcing evidence that ADHD characteristics and symptom severity directly predict lower subjective well-being [7,57,58]. Moreover, EFs, particularly metacognitive abilities, appear to influence QoL beyond symptom levels [29,31], supporting previous reports that everyday metacognitive processes mediate the relationship between ADHD and perceived life quality [59]. Given the potential conceptual overlap between these constructs, these results should be interpreted as reflecting associative, rather than causal, relationships among group membership, organization-in-time abilities, executive functioning, and QoL.

In addition to the contributions of the group and metacognitive abilities, organization-in-time domains accounted for unique variances in QoL. The association between QoL and emotional responses to time-organization impairments (TOPS Domain C) can be understood in light of prior findings linking emotional ability with functional impairment, and functional impairment with poor QoL among adults with ADHD [37,48,60]. Similarly, the predictive role of daily time-organization performance (TOPS Domain B) may reflect the sense of control derived from successfully managing time and daily tasks [6]. These results are also consistent with previous findings identifying inhibition (a specific metacognitive ability) and emotional responses to organization-in-time impairments (TOPS Domain C) as predictors of QoL for adults with SLD [6].

Together, these findings advance understanding of how specific executive and time organization abilities jointly shape adults’ perceived QoL. This perspective aligns with broader evidence suggesting that reduced QoL in ADHD may be explained, at least in part, by daily functional difficulties [61,62]. Weiss [63] emphasized that rather than mere symptom reduction, functional improvement and QoL should be regarded as primary outcomes in ADHD research and intervention. The present study supports this view by identifying metacognitive EFs and organization-in-time abilities as key functional mechanisms linked to adults’ daily well-being.

### 4.5. Limitations and Future Directions

These findings advance the understanding of functional and cognitive mechanisms underlying QoL in adults with ADHD; however, several methodological limitations should be acknowledged. Although the 2017–2018 dataset predates the *DSM-5-TR* (2022), the findings remain relevant as the core diagnostic criteria for adult ADHD have remained fundamentally consistent with the *DSM-5*. Since the updates were descriptive rather than diagnostic, the study sample reflects current standards. Furthermore, the chronic nature of ADHD ensures the stability of the diagnosis and its functional implications (e.g., executive and time-organization difficulties) over time, supporting the continued relevance of the findings to current clinical framework. The study relied solely on self-reports, which capture ecologically valid experiences but may introduce perception bias. Consequently, the findings reflect participants’ subjective evaluation rather than objective performance, which limits the generalizability of the results regarding objective functional status. The inclusion of participants with comorbid neurodevelopmental or psychiatric conditions reflects real-world ADHD heterogeneity but may have confounded some associations. The cross-sectional design also limits causal interpretation. Although the sample size was modest, an a priori power analysis confirmed adequate statistical power; still, replication with larger and more diverse samples is warranted to strengthen generalizability and clarify how EFs, organization-in-time abilities, and QoL interact in adults with ADHD.

Future studies should adopt multimethod approaches that combine self-report and objective assessments to strengthen the validity of findings and enhance generalizability. Further research is also needed to deepen the understanding of organization-in-time abilities, their expression in adults’ daily functioning, and their contribution to QoL. In addition, exploring how other ADHD-related features, such as emotional regulation, jointly influence QoL may clarify the complex mechanisms underlying daily functioning in this population.

## 5. Conclusions

In sum, the present study broadens the understanding of adults with ADHD by demonstrating that difficulties in EFs and organization-in-time abilities are interrelated and jointly contribute to their reduced QoL. These findings extend previous evidence among adults with SLD [6], emphasizing that similar associations also exist within the ADHD population. Using ecologically valid self-report measures enabled the study to capture participants’ subjective perceptions within their daily contexts, reflecting the real-life cognitive and emotional challenges they experience and manage. This approach strengthens the ecological validity of the findings and enhances the relevance of the results to everyday functioning.

From a clinical perspective, recognizing the interrelations between metacognitive abilities, emotional responses following time disorganization, and daily time-organization performance highlights key mechanisms underlying functional and emotional difficulties in adults with ADHD. Understanding these mechanisms may guide the development of more focused and ecologically grounded interventions to improve daily participation and QoL. Together, the findings outline a conceptual framework linking executive and temporal organization abilities with QoL, offering a foundation for future studies integrating objective and longitudinal approaches.

## Figures and Tables

**Table 1:** Means and standard deviations of the Time Organisation and Participation Scale (TOPS) domain scores.

TOPS Domain	ADHD Group (*n* = 68)	Control Group (*n* = 52)	*F*	η^2^
	*M* (*SD*)			
A (pace)	3.24 (0.84)	4.46 (0.57)	82.44 ***	0.41
B (performance)	2.53 (0.86)	4.15 (0.51)	143.24 ***	0.55
C (emotional response)	3.03 (0.58)	4.05 (0.70)	76.60 ***	0.39

Note. ADHD = attention-deficit/hyperactivity disorder. *** *p* < 0.001; lower score = lower performance.

**Table 2:** Means and standard deviations of the Adult ADHD Quality of Life Questionnaire (AAQoL) total and subscales scores.

AAQoL Total and Subscale	ADHD Group (*n* = 66)	Control Group (*n* = 53)	*F*	η^2^
	*M* (*SD*)			
Life productivity	52.25 (13.47)	85.42 (10.27)	218.76 ***	0.65
Psychological health	57.32 (17.31)	80.12 (13.31)	62.68 ***	0.35
Life outlook	62.42 (15.94)	78.33 (11.69)	38.57 ***	0.25
Relationships	61.21 (16.06)	82.34 (11.08)	66.32 ***	0.36
Total score	58.00 (12.37)	81.93 (9.45)	*t* = −11.81 ***	Cohen’s *d* effect size = 2.17

Note. *** *p* < 0.001.

**Table 3:** Prediction of the AAQoL total score by group membership, BRIEF-A index scores, and TOPS subscales scores.

Model	AAQoL Total Score		
	*B*	*SE*	β
1	*R*^2^ (adj *R*^2^) = 0.52 (0.51); *F* change = 127.19 ***		
Group	−3.18	0.28	−0.72 ***
2	*R*^2^ (adj *R*^2^) = 0.15 (0.66); *F* change = 53.52 ***		
Group	−0.18	0.47	−0.04 ***
Emotional responses (TOPS C)	1.40	0.19	0.51 ***
3	*R*^2^ (adj *R*^2^) = 0.09 (0.75); *F* change = 43.49 ***		
Group	0.42	0.41	0.09
Emotional responses (TOPS C)	1.11	0.17	0.41 ***
Metacognitive index (BRIEF-A)	−0.08	0.01	−0.64 ***
4	*R*^2^ (adj *R*^2^) = 0.02 (0.77); *F* change = 9.04 **		
Group	0.45	0.40	0.10
Emotional responses (TOPS C)	1.03	0.16	0.37 ***
Metacognitive index (BRIEF-A)	−0.06	0.01	−0.47 ***
Performance (TOPS B)	0.48	0.16	0.24 **

Note. BRIEF-A = Behavior Rating Inventory of Executive Function-adult version. *** *p* < 0.001, ** *p* < 0.01.

## Data Availability

The data are available on request from the corresponding author (S.R.). The data are not publicly available due to ethical and privacy restrictions.

## Abbreviations

The following abbreviations are used in this manuscript:

AAQoL	Adult ADHD Quality of Life Questionnaire
ADHD	Attention-deficit/hyperactivity disorder
ANOVA	Analysis of variance
ASRS	Adult ADHD Self-Report Scale
BADDS	Brown Attention-Deficit Disorder Scales
BRI	Behavioral-regulation index (BRIEF-A)
BRIEF-A	Behavior Rating Inventory of Executive Function-Adult version
*DSM-5-TR*	*Diagnostic and Statistical Manual of Mental Disorders*, 5th ed., text revision
EF	Executive function
GEC	Global executive composite (BRIEF-A)
MANOVA	Multiple analyses of variance
MI	Metacognition index (BRIEF-A)
QoL	Quality of life
SLD	Specific learning disabilities
TOPS	Time Organisation and Participation Scale
